# Contributing Factors and Outcome after Cryotherapy of Molluscum Contagiosum among Patients Attending Tertiary Hospital, Northern Tanzania: A Descriptive Prospective Cohort Study

**DOI:** 10.1155/2021/9653651

**Published:** 2021-07-01

**Authors:** Peter J. Chapa, Daudi R. Mavura, Rune Philemon, Lulyritha Kini, Elisante J. Masenga

**Affiliations:** ^1^Department of Dermatovenereology, Kilimanjaro Christian Medical University College, P. O. Box 2240, Moshi, Tanzania; ^2^Department of Dermatovenereology, Kilimanjaro Christian Medical Centre, P.O Box 3010, Moshi, Tanzania; ^3^Department of Paediatrics and Child Health, Kilimanjaro Christian Medical Centre, P. O. Box 2240, Moshi, Tanzania

## Abstract

**Background:**

Molluscum contagiosum (MC) is a benign infection caused by a member of the Poxviridae family, molluscum contagiosum virus (MCV). The contributing factors for MCV infection are different in different populations and study areas. Few studies have been conducted to determine the effectiveness of cryotherapy in the treatment of MC. The study's objectives were to determine contributing factors and outcome after cryotherapy of MC among patients attending a tertiary hospital in Northern Tanzania.

**Methods:**

A hospital-based cohort study was conducted at the Regional Dermatology Training Centre (RDTC) from September 2018 to August 2019, involving all patients clinically diagnosed with MC. We used a consecutive sampling method to recruit study participants. We treated all participants with cryotherapy and assessed them after two weeks. Data were collected using a structured questionnaire and analyzed using Statistical Package for the Social Sciences (SPSS) software version 21.

**Results:**

There were 49 patients with MC who agreed to participate in this study with a median age of 8 (IQR 3–22). We found 18.4% of patients with active atopic dermatitis (AD) had MC while those with a history of atopic diseases (Ad) were 32%, and 22.4% had a history of using immunosuppressive drugs. The clearance rate of cryotherapy on MC lesions was found to be 94%. Hypopigmentation was the commonest adverse effect.

**Conclusion:**

The findings of this study show that AD and immunosuppression may be contributing to MC development. Based on the clearance rate results, cryotherapy has shown to be effective and may be used in the treatment of MC.

## 1. Introduction

Molluscum contagiosum (MC) is a benign infection caused by a member of the Poxviridae family, molluscum contagiosum virus (MCV) [1, 2]. Since eradicating smallpox, MCV has been the principal poxvirus cause of human disease [3].

The estimated worldwide prevalence of the MCV infection ranges from 5.0% to 7.5% [4]. The prevalence is increased in immunosuppressed individuals (5.0–18%) [5]. There is an increased incidence of atopic dermatitis (AD) in the developing world, which has been documented to be contributing to the development of MC. There are conflicting results in different studies on whether AD or immunosuppression contributes to the development of MC. There is also insufficient evidence to suggest the superiority of any particular treatment option, and recent publications revealed that it is difficult to advocate one single treatment above others [6, 7]. To determine the contributing factors and outcome after cryotherapy of MC, we conducted a study of all patients who attended the Regional Dermatology Training Centre (RDTC) clinic during the study period with MC diagnosed clinically.

## 2. Materials and Methods

This study was a hospital-based descriptive prospective cohort study conducted at the Regional Dermatology Training Centre (RDTC) in Moshi, Tanzania, from September 2018 to August 2019. RDTC is located at the Kilimanjaro Christian Medical Centre (KCMC) Hospital compound in Moshi Kilimanjaro, the northern zone of Tanzania. The hospital lies at the foot of mountain Kilimanjaro.

All patients who attended RDTC clinic during the study period with MC diagnosed clinically with single or multiple umbilicated papules measuring 2 to 5 mm in diameter were included. The study excluded all patients who received treatment for the last two weeks. Ethical clearance was obtained from the Kilimanjaro Christian Medical University College Research and Ethics Review Committee. Those diagnosed with MC were enrolled consecutively and consented and assented to participate in the study. We collected data using a structured questionnaire. For patients whose HIV status was unknown, they were counselled to permit screening for their serostatus. Treatment with cryotherapy using liquid nitrogen was given to patients at the beginning of the study and, if necessary, after two weeks. Cryotherapy was employed with a spraying gun in 6 to 10 seconds at a 2 cm distance from the lesions. The outcome of cryotherapy was assessed by examining the disappearance of lesions exposed to liquid nitrogen for two visits.

Data were double entered, cleaned, validated, and analyzed using SPSS version 21. Descriptive analysis was done. Categorical variables were summarized using frequency and proportions, while continuous variables were summarized using central tendency measures with their respective measures of dispersion.

## 3. Results

There were 49 participants with MC in this study; their ages ranged from 1 year to 51 years, with a median age of 8 (IQR 3–22). The majority of participants, 73.5% (36), were aged below 18 years. Females comprised 61.2% (30) and 67.3% (33) were from urban areas. Most patients had MC lesions of less than 12 weeks duration, and 71.4% (27) and 73.5% (36) had less than 15 lesions, as shown in Table 1.

**Figure 1:**
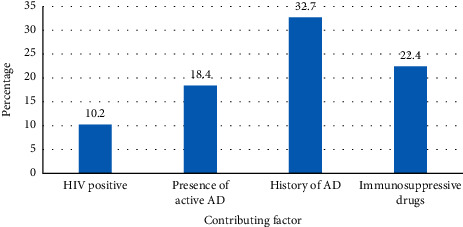
Contributing factors of molluscum contagiosum (*N* = 49).

Among 49 participants who were diagnosed with MC, the prevalence of a history of atopic diseases (Ad) was 32.7% (16), history of using immunosuppressive drugs 22.4% (11), active atopic dermatitis (AD) 18.4% (9), and 10.2% (5) were HIV positive, Figure 1.

**Figure 2:**
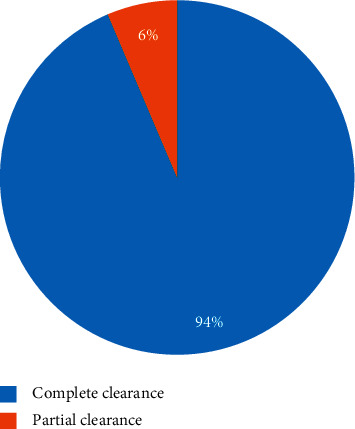
Overall clearance rate of molluscum contagiosum at the end of the second visit (*N* = 47).

All forty-nine (49) participants were treated with cryotherapy. Two (2) patients were lost to follow up. The remaining 47 patients completed the cryotherapy treatment. 53.2% (25) participants needed the 2^nd^ round of cryotherapy treatment. Overall, the 100% clearance rate at the end of the 2^nd^ visit was 93.6% (44), as indicated in Figure 2.

Pain during spray or application was the most frequently reported adverse effect of cryotherapy as 89.4% (42) patients experienced it. Other commonest adverse effects reported are hypopigmentation 70.2% (33), hyperpigmentation 38.3% (18), and blistering 36.2% (17), Table 2.

## 4. Discussion

This descriptive prospective cohort study provides information on the contributing factors and outcome after cryotherapy of MC. In this study, 18.4% of the participants were diagnosed with active AD at the MC diagnosis. Similar findings were observed in a study done in Greece, and the USA that showed 18.4% and 24.2%, respectively, had AD at the time of diagnosis of MC [8, 9]. This could be explained by the fact that both studies were conducted in similar a setting that is a dermatology clinic where the diagnosis of AD and MC is made by a dermatologist. MCV is also considered as one of the cutaneous manifestations of immunosuppressive conditions including AD which causes skin barrier disruption. A different result was seen in a study conducted in Spain, where the percentage of patients with MC and AD was reported as high as 49% [10]. In our study, 32.7% had a history of atopic diseases. A study conducted in the USA revealed a similar finding that 37% had a history of atopic diseases [11].

Cryotherapy is one of the most commonly used methods for the treatment of MC. Liquid nitrogen, which boils at −96°C, is the most effective cryogen for clinical use. The rapid development of intracellular ice crystals produces shearing and rupture of cell membranes, organelles, and the cytoskeleton. Tissue damage also occurs because ice crystallization extracts free water from the intracellular solutes, resulting in protein denaturation. These complex mechanisms of cell death are further enhanced by damage to the microvascular circulation, which results in secondary anoxia and hemorrhagic necrosis. In this study, 47 participants were treated with cryotherapy, and the complete clearance rate was 94%, with pain, pigmentary changes, and blistering as the most common adverse treatment effects. Similar findings were reported in studies done in Kuwait and Iran, where the complete clearance rate was 100% and 93.3%, respectively [12, 13]. Cryotherapy has several advantages in the treatment of MC. The preparation time is short, requires no expensive supplies.

## 5. Conclusion

The results of this study have provided valuable information on the contributing factors and outcome of cryotherapy in patients with MC. The percentage of patients with AD and MC was 18.4%, and 22.4% had a history of using immunosuppressive drugs. Few patients were found to be HIV positive. These findings show the possibility of the contribution of AD and immunosuppression in the development of MC. Further studies are recommended to look at the causal relationship of MC and AD or immunosuppression. The clearance rate of cryotherapy on MC lesions was found to be 94%. On this basis of the results, cryotherapy has shown to be effective and maybe a preferred treatment option for MC, especially in developing countries.

## Figures and Tables

**Table 1:** Sociodemographic characteristics of participants (*N* = 49).

Characteristic	*n*	%
Age		
<18	36	73.5
≥18	13	26.5

Sex		
Male	19	38.8
Female	30	61.2

Residence		
Urban	33	67.3
Rural	16	32.7

MC duration in weeks		
1.0–12	27	71.4
13–36	12	8.2
37–52	10	20.4

Number of lesions		
<15	36	73.5
15–30	11	22.4
>30	2	4.1

**Table 2:** Adverse effects of cryotherapy (*N *=* *47).

Adverse effect	*n*	%
Pain		
Yes	42	89.4
No	5	10.6

Blistering		
Yes	17	36.2
No	30	63.8

Erosion		
Yes	1	2.1
No	46	97.9

Scarring		
Yes	4	8.5
No	43	91.5

Hypopigmentation		
Yes	33	14
No	70.2	29.8

Hyperpigmentation		
Yes	18	38.3
No	29	61.7

## Data Availability

Data are available and will be submitted upon request.

## List of abbreviations

AD: Atopic dermatitis
MC: Molluscum contagiosum
MCV: Molluscum contagiosum virus
RDTC: Regional Dermatology Training Centre
SPSS: Statistical Package for the Social Sciences.
